# Safety and tissue remodeling assay of small intestinal submucosa meshes using a modified porcine surgical hernia model

**DOI:** 10.1038/s41598-023-50425-5

**Published:** 2024-01-03

**Authors:** Chenghu Liu, Zhenhua Lin, Wenting Ruan, Xiaoxiao Gai, Qiujin Qu, Changbin Wang, Fuyu Zhu, Xiaoxia Sun, Jian Zhang

**Affiliations:** 1 Institute of Immunopharmacology and Immunotherapy, School of Pharmaceutical Sciences, Shandong University, 44 Wenhua Xi Rd, Jinan, 250012 Shandong China; 2 Shandong Institute of Medical Device and Pharmaceutical Packaging Inspection, NMPA Key Laboratory for Safety Evaluation of Biomaterials and Medical Devices, Jinan, 250101 China

**Keywords:** Tissue engineering, Molecular medicine

## Abstract

In studies to date, meshes based on extracellular matrix (ECM) have been extensively used in clinical applications. Unfortunately, little is known about the function of the immunogenic residual, absorbable profile during the tissue repair process. Moreover, there needs to be a recognized preclinical animal model to investigate the safety and efficacy of extracellular matrix meshes. Herein, we designed and fabricated a kind of SIS mesh followed by a scanned electron micrograph characterization and tested α-Gal antigen clearance rate and DNA residual. In order to prove the biocompatibility of the SIS mesh, cell viability, chemotaxis assay and local tissue reaction were assessed by MTT and RTCA cytotoxicity test in vitro as well as implantation and degradation experiments in vivo. Furthermore, we developed a stable preclinical animal model in the porcine ventral hernia repair investigation, which using laparoscopic plus open hybridization method to evaluate tissue adhesion, explant mechanical performance, and histologic analysis after mesh implantation. More importantly, we established a semi-quantitative scoring system to examine the ECM degradation, tissue remodeling and regeneration in the modified porcine surgical hernia model for the first time. Our results highlight the application prospect of the improved porcine ventral hernia model for the safety and efficacy investigation of hernia repair meshes.

## Introduction

With the development of material science and tissue engineering technique, extracellular matrix (ECM) materials have been widely available in clinical practice, such as hernia repair^1,2^, breast reconstruction and orthopedic substitution^3,4^. It has been reported that there are about 20 million hernia patients annually worldwide, and about 80% of them need to mesh repair surgery^5,6^. Among these, ECM based mesh repair has been practically developed for hernia treatment^7,8^, especially the laparoscopic technology that has emerged over the past several decades. ECM mesh can be obtained from animal small intestinal submucosa (SIS), pericardium, dermis, urinary bladder and other tissue sources via chemical, enzymatic, and physical decellularization processes^9,10^. As such, the approved decellularized SIS meshes are welcomed in regenerative medicine applications due to their absorbable, low immunogenic and analogous microstructure character, which includes proteins, proteoglycans, and growth factor remained for tissue remodeling^11^. Remarkably, most decellularized SIS meshes also have excellent biocompatibility and degradable performance related to host tissue repair and remodeling.

However, many recent reports indicated that recurrence had remained at a higher percentage. The adhesion and fistula formation, coupled with other mesh-related complications after hernia repair has been reported frequently^12–16^. Zheng et al. have reported that one kind of porcine small intestine submucosa commercially available is not an appropriate acellular biomaterial since it is able to cause variable inflammatory responses after implantation^17^. Though DNA and α-Gal residua after decellularization have been concerned extensively^18–20^, there is limited knowledge about the effect of DNA and α-Gal antigen residua, dynamic balance between material degradation and tissue remodeling during the mesh-induced tissue repair process in a stable animal models.

Therefore, the lack of recognized animal models can be used to evaluate the safety and repair effect, hindering the development and clinical application of new ECM materials^21^. In this study, we f developed a decellularized SIS mesh and performed the DNA and α-Gal antigen residual determination to identify the immunogenicity potential. Moreover, we performed the biocompatibility of the SIS mesh based on cytotoxicity with MTT and real-time cell analysis (RTCA) method, and the Balb/c 3T3 cell chemotaxis assay and implantation degradation assay to investigate the feasibility of these meshes using a collagen composited polyester mesh as a control. Furthermore, we developed a useful semi-quantitative scoring system in a modified porcine ventral hernia repair model to evaluate the tissue remodeling and anti-adhesion effect, including tissue ingrowth, integration, degradation, and host ECM deposition.

## Materials and methods

### Materials

Decellularized porcine Small Intestinal Submucosal (SIS) matrix (4 layers) was prepared using the established techniques. Briefly, the small intestines were harvested from pigs following the euthanasia in the morning, then the SIS was decellularized by agitation in a solution containing 1 M NaCl and 0.3% w/w Triton-X 100 for 18 h for decellularization. The decellularized SIS sample was treated with 0.10% peracetic acid solution followed by a washing process with 1 × PBS solution (pH = 7.4) containing 5 mm EDTA. The 4 layer decellularized SIS matrix with vertical compression was obtained from the delipidization and sterilization treatment with isopropanol. The composite meshes were commercially obtained from Sofradim Production (France) and used as marketed controls.

### Animals

Eighteen New Zealand female white rabbits weighing 2.5–3.5 kg were purchased from Jinan Jinfeng animal Co., Ltd (SCXK (Lu) 20180006), China and acclimated to the clean grade facility (temperature: 17–25 ℃, relative humidity: 40–70%, light cycle: 12 h light and 12 h dark, alternately) for 7 days. A total of 12 female Yucatan minipigs weighing 40–50 kg were purchased from Tianjin Bainong Experimental Animal Breeding Technology Co., Ltd (SCXK (Jin) 2020-0002), China and acclimated to the conventional facility for 7 days (temperature: 16–28 ℃, light cycle: 12 h light and 12 h dark, alternately). All animals were housed, fed, handled and utilized in accordance with the protocols approved by Shandong Institute of Medical Device and Pharmaceutical Packaging Inspection. The experiments were conducted according to the approved “Guide for the Care and Use of Laboratory Animals” of the institute.

### Mesh characterization

The surface structural micrographs of the meshes were recorded with scanning electron micrographs (SEM, Hitachi SU8010). Briefly, prepare the 1 cm × 1 cm samples and mount them on aluminum stubs using silver paint for SEM imaging.

### Animal model preparation

A model of ventral hernia repair was established following the laboratory standard operating procedure. Briefly, all animals were fasted for at least 12 h prior to surgery. After sedation, animals were intubated and maintained under anesthesia with 0.5–2% isoflurane. The animals were placed in dorsal recumbency, and the ventral abdomen was prepared using the aseptic surgery technique. A midline incision (~ 5 cm) with 2 cm diameter muscular defects to perform aponeurosis defects of the abdominal wall in the surgical area. Suture the skin and subcutaneous tissue using a 3-0 surgical suture. Observe the abdominal wall protrusion of animals every day. Measure the size of the hernia ring and confirm the success of the abdominal wall hernia model by hernia content palpation. Herniorrhaphy should be conducted after the edema of the abdominal wall defect area is eliminated. Measure the diameter of the hernia ring and record as long axis × minor axis (millimeter) before repair.

### Surgical repair procedure

After 90 days of surgery, all animals developed hernias. The Laparoscopic (Olympus CLV-S190) with Open Hybridization method was used for hernia mesh repair. Select the appropriate meshes according to the size of the hernia ring. In order to completely cover the defect, the hernia ring defect was taken as the center of the mesh 5 cm beyond the edge of the defect area. Additionally, the screwed nails were utilized to anchor the repair mesh, and all incisions were closed by a 3-0 stitch. Postoperative antibiotic prophylaxis was provided for 7 days. The abdominal region of each animal was examined daily to assess the condition of the wound and healing. The wound healing level was determined according to the cleaning degree and healing level of the incision. Animal blood samples were collected to measure the blood routine, blood coagulation and blood biochemistry for pre-operation, 2 weeks and 1 month after the repair operation.

### DNA residual determination

The residual DNA of the mesh was analyzed using PrepSEQ™ Sample Preparation Kits (Thermo FisherScientific, Waltham, MA) according to the manufacturer’s instructions. In brief, the DNA sample was digested with fresh preparing lysis solution and bound to Magnetic Particles that preheat at 37 ℃. After the samples were twice washed from the magnetic stand, eluted the DNA residue with 50 µl of Elution Buffer. The residual amounts were detected by Quant-iT™ PicoGreen™ dsDNA Reagent and Kit (Thermo FisherScientific, Waltham, MA).

### α-Gal antigen assay

The gal-antigens in the meshes were detected by incubating overnight with a mouse monoclonal anti-Gal IgM antibody (M86) at 100 times dilution, to bind to the gal epitope from the experimental samples. ELISA was used to measure the remaining antibody in the supernatants, the α-gal-bovine serum albumin was utilized as a solid-phase antigen, and horseradish peroxidase-conjugated goat anti-mouse IgM antibody as a secondary antibody. The absorbance was read at 450 nm using a microplate reader.

### In vitro cytotoxicity assay using MTT and RTCA method

Briefly, the vigorous L929 mouse fibroblast cells (Conservation Genetics CAS Kunming Cell Bank, China) were incubated with the extract of meshes which were prepared, based on the ratio of 3 cm^2^/ml, with the condition of 60 rpm at (37 ± 1) °C for (72 ± 1) h in a humidified atmosphere under 5% CO_2_ at 37 °C. After 72 h incubation, the cell morphology was observed and evaluated using an optical microscope (Olympus IX71, Japan) according to Table 1. Then the 50 µl of MTT (Sigma, MKBH9792V) solution was added into each test well followed by further incubated for 2 h in the incubator at 37 °C. The absorbance was measured with the microplate reader (Thermofisher Multiskan MK3, USA) at the wavelength of 570 nm (reference wavelength 650 nm) after adding 100 µl of isopropanol (Sinopharm Chemical Reagent Co., Ltd., 20180530) into each well, and the reduction of viability was calculated using the following equation Viab.% = 100 × OD570_e_/OD570_b_, where OD570_e_ and OD570_b_ is the mean value of the measured optical density of the test sample and the blanks, respectively. If viability is reduced to < 70% of the blank, it has a cytotoxic potential.

**Table 1:** Qualitative morphological grading of cytotoxicity.

Grade	Reactivity	Conditions of all cultures
0	None	Discrete intracytoplasmatic granules, no cell lysis, no reduction of cell growth
1	Slight	Not more than 20% of the cells are round, loosely attached and without intracytoplasmatic granules, or show changes in morphology; occasional lysed cells are present; only slight growth inhibition observable
2	Mild	Not more than 50% of the cells are round, devoid of intracytoplasmatic granules, no extensive cell lysis; not more than 50% growth inhibition observable
3	Moderate	Not more than 70% of the cell layers contain rounded cells or are lysed; cell layers not completely destroyed, but more than 50% growth inhibition observable
4	Severe	Nearly complete or complete destruction of the cell layers

The Real Time Cell Analysis (RTCA) test has been previously described^22^. In brief, after the cell index (CI) background value of the medium was measured, different groups of L929 cell suspensions were prepared with 10^5^ cells/ml. Then 30 min before starting the software schedule, 300 μl cell suspension was added to the E-plate for real-time and dynamic cell proliferation detection. After 24 h, the old culture medium was replaced with extracts and the experiment was run for another 48 h. The impedance signals were recorded every 1 h until the end of the experiment. The CI value was given by the RTCA software package based on the impedance signal.

### Chemotaxis assay

Briefly, SIS mesh was cut into pieces and digested with pepsin (1 mg/ml) in 0.01 M HCl (pH 2.0) and was then centrifuged at 25,000*g* for 30 min to obtain SIS pellet and supernatant, respectively. All materials were stored at − 80 °C until use. Balb/c 3T3 cells were plated and grown to 80–90%, which transferred to DMEM (containing 0.5% heat inactivated FBS) for overnight culture. Cells were trypsinized and resuspended in ordinary DMEM, and 30,000 cells were loaded into the top well of the chemotaxis chamber (1426.65 ± 11.59 μg/ml SIS hydrogel, 967.58 ± 52.60 μg/ml SIS pellet, and 463.15 ± 31.55 μg/ml SIS Supernatant), placing the test chamber into incubator containing 5% CO_2_ at 37 °C for 3 h. Migrated cells were stained with DAPI and observed under a microscope.

### Implantation and degradation in vivo

The rabbit subcutaneous implantation test was conducted to evaluate the local tissue reaction effect for the mesh. The procedures were conducted according to the established protocol. Briefly, animals were anesthetized by intravenous pentobarbital sodium dose. The surgical site was wiped with a tincture of iodine and 75% alcohol. High Density Polyethylene (HDPE, Hatano Research Institute, Japan) negative control and mesh samples of 10 mm × 10 mm were implanted into the subcutaneous tissue along each side of the spine, which 2.5 cm from the midline and parallel to the spinal column, and about 2.5 cm apart from each other. The incisions were closed using 3-0 stitch. Animals were euthanized after 1 w, 4 w and 13 w implantation and the implanted specimen and enough adjacent normal tissues were excised and prepared for degradation and histopathologic analysis. For local effects after implantation, the average score for the control treatment is subtracted from the test treatment average to determine a reactivity grade.

### Adhesion evaluation

The adhesion degree and location of different groups were observed and recorded. The adhesion ratio was calculated as the following formula: P = $\frac{{{\text{A}}}_{1}}{{\text{A}}}$×100%, where: P represents the adhesion ratio, A1 represents adhesion area, and A represents the total contact area between tissue and mesh. The adhesion degree including adhesion strength and adhesion area between intraperitoneal organs and meshes was evaluated^23–26^_._

### Mechanical testing

The tensile strength of the composite mesh (n = 18) and the porcine SIS mesh (n = 18) were determined by the tensile testing machine (Instron, USA). Three samples of 10 mm × 50 mm were prepared for each explant. The maximum load sustained by the test sample was recorded in Newtons (N). The tensile strength per unit was recorded as Newtons per centimeter (N/cm).

### Histologic analysis

Explant samples (about 10 mm × 10 mm) were collected and stained with H&E. A pathologist conducted histologic observation and evaluation using high-powered light microscopy (40 ×, 100 ×, and 200 × magnification). Cellular infiltration, neovascularization, ECM degradation, tissue ingrowth, integration and host ECM deposition were recorded and scored according to the semi-quantitative scoring system to evaluate the tissue repair effect based on Table 2.

**Table 2:** Semi-quantitative scoring system of repair area.

Response	Score				
	0	1	2	3	4
Neovascularisation	0	Minimal capillary proliferation, focal, 1 to 3 buds	Groups of 4 to 7 capillaries with supporting fibroblastic structures	Broad band of capillaries with supporting fibroblastic structures	Extensive band of capillaries with supporting fibroblastic structures
Fatty infiltrate	0	Minimal amount of fat associated with fibrosis	Several layers of fat and fibrosis	Elongated and broad accumulation of fat cells about the implant site	Extensive fat completely surrounding the implant
Fibrosis	0	Narrow band	Moderately thick band	Thick band	Extensive band
Integration	0	Poor combination of abdominal wall tissue and scaffold	Partial combination of abdominal wall tissue and scaffold	Abdominal wall tissue is mostly combined with scaffold	Total combination of abdominal wall tissue and scaffold
Cellular infiltration	0	Cell contact with scaffold surface, Not immersed in the scaffold	Cell immersed in the scaffold, But not to the central area	Cell immersed into the central area of the scaffold	Totally immersed
Tissue ingrowth	0	Poor tissue ingrowth, sparse	Multifocal inward growth	Consistent inward growth	Total ingrowth
ECM deposition	0	Host ECM deposits on the scaffold surface	Host ECM deposits in the scaffold, but not to the central area	Host ECM deposits in the scaffold, including the central area	Total deposition
Scaffold degradation	0	Partial scaffold degradation, layered by cells, blood vessels, host tissues, etc.	Major scaffold degradation, structural disintegration	Severe scaffold degradation, difficult to distinguish from host tissue	Scaffold completely degraded

### Statistical analysis

Statistical analysis was performed using a student test. A *p-*value < 0.05 was considered statistically significant. Data are presented as the mean ± SD.

### The ethics statement

This study and included experimental procedures were approved by the institutional animal care and use committee of Shandong Institute of Medical Device and Pharmaceutical Packaging Inspection (approval No. KY2023004). All animal housing and experiments were conducted in strict accordance with the approved “Guide for the Care and Use of Laboratory Animals and also with arrive guidelines” of the institute.

## Results

### Electron microscopy imaging

**Figure 1:**
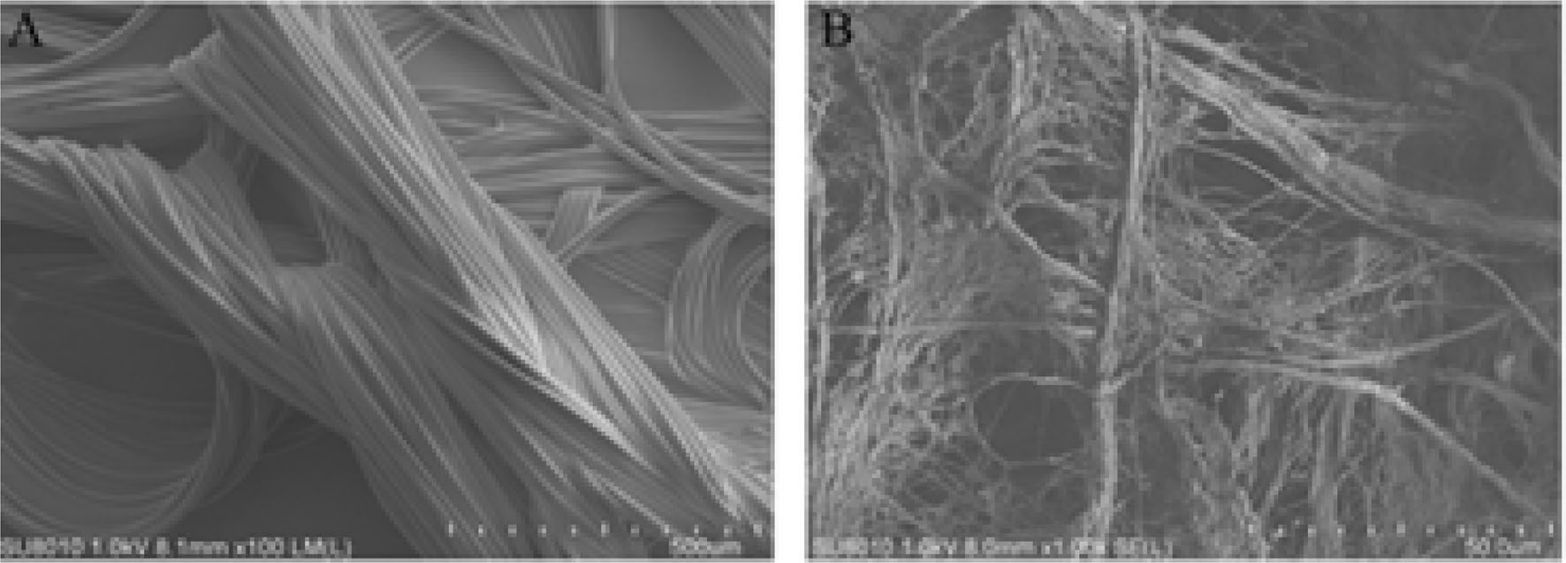
(**A**) SEM imaging of the composite mesh (scale bar, 500 μm). (**B**) SEM imaging of the SIS mesh (scale bar, 50 μm).

As shown in Fig. 1A,B, the polyester fibril is tightly bound to the collagen film for the composite mesh, whereas the SIS mesh displayed a porous surface.

### Results of DNA residual assay

**Figure 2:**
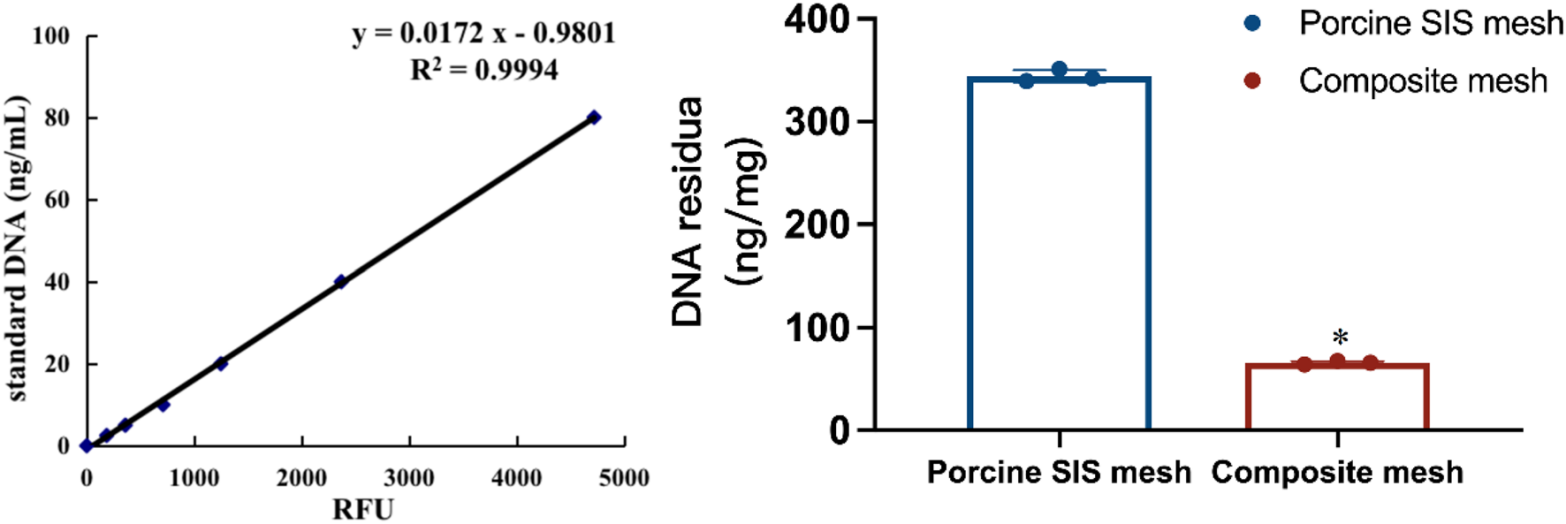
The DNA residua in the porcine SIS mesh and composite mesh (*p < 0.05).

Seven serial dilutions of the lambda DNA were tested with technical triplicate to estimate the residual DNA of the acellular porcine SIS mesh using lambda DNA as a standard. The standard curve of lambda DNA exhibited excellent linearity (R^2^ = 0.9994) with a linear correlation formula y = 0.0172 x—0.9801 (see Fig. 2). According to the formula of the standard curve, the DNA residual in the acellular porcine SIS mesh and the composite mesh was 344.24 ± 6.06 ng/mg and 65.76 ± 1.54 ng/mg, respectively.

### Analysis of α-Gal antigen clearance

**Figure 3:**
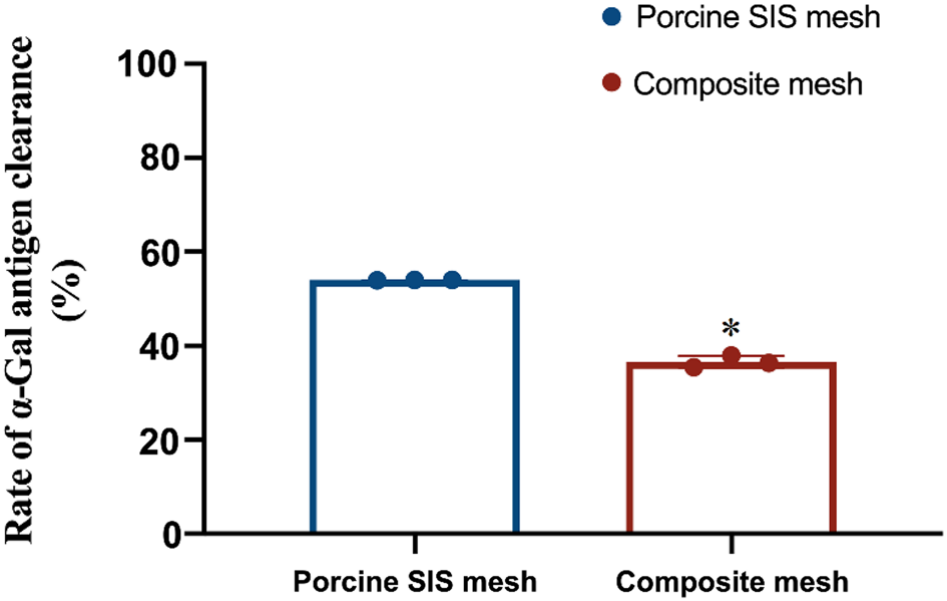
The α-Gal antigen clearance of porcine SIS mesh and composite mesh (*p < 0.05).

Alpha-gal antigens are glycoprotein and glycolipid antigens present on mammalian cell membranes. This assay calculates clearance of α-gal antigen by comparing α-gal antigen content in samples to be tested before and after treatment. 1% Bovine serum albumin was utilized as the standard, which is double diluted from 20 to 1.25 μg/μl, followed by obtaining the logarithmic correlation of the standard concentration R^2^ = 0.99 through the logarithmic relationship (see Fig. 3). The α-gal antigen clearance was obtained according to the standard curve and the α-gal scavenging ability of its starting material. In contrast, the rate of α-gal antigen clearance is 54.01% ± 0.02% and 36.60% ± 1.27% for acellular porcine SIS mesh and the composite mesh, respectively, which indicates the existence of evidenced cellular component in the mesh.

### In vitro cytotoxicity assessment by the MTT and RTCA method

**Figure 4:**
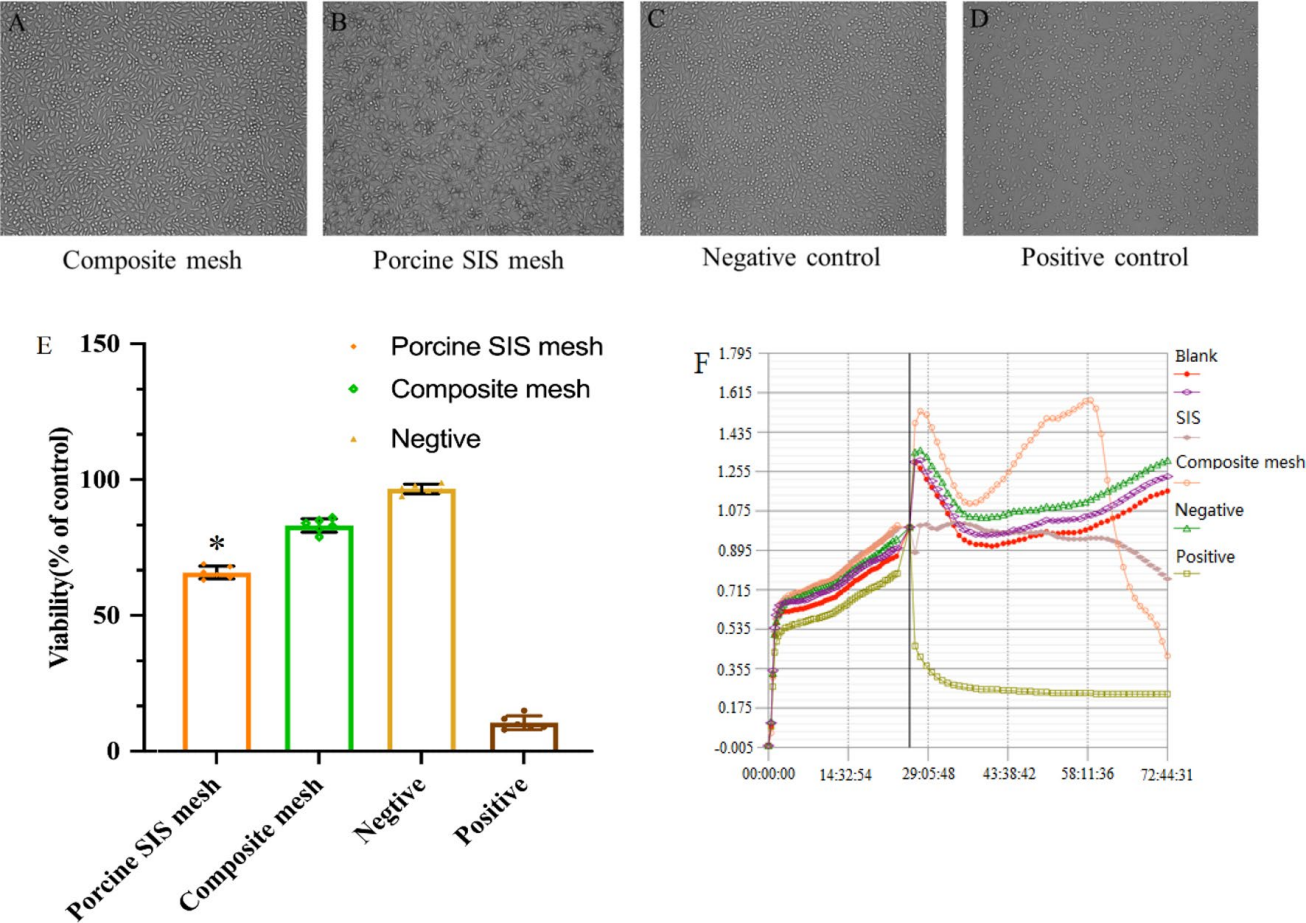
L929 cytotoxicity after culture with the extract for 72 h; (**A**) composite mesh and (**B**) porcine SIS mesh groups, and (**C**) negative and (**D**) positive control groups. (**E**) The average cell viability of L929 cell line with Composite meh and Porcine SIS mesh. (**F**) The dynamic cell index of extracts prepared from Composite control mesh and Porcine SIS mesh during 48 h incubation. Data represent mean ± SD; *p < 0.05. The data presented here represent a total of three independent replicates.

The in vitro cytotoxicity was investigated using the MTT and RTCA method, respectively. As shown in Fig. 4A–D, a small amount of the cells were round, loosely attached. The cell shows the morphology changes in the SIS mesh extract groups, which indicated a slight inhibition effect compared to the composite mesh as their cell viability was 65.8% ± 2.3% and 86.7% ± 2.9%, respectively (Fig. 4E) regarding the tested L929 mouse fibroblast cell line. In the meantime, most cells were normal in morphology, a small amount of the cells are round, loosely attached, and without changes in morphology in the negative control group (HDPE, Hatano Research Institute, Japan). The cell layers in the positive control group (ZDEC polyurethanes, Hatano Research Institute, Japan) were completed destruction of the cell layers. The qualitative morphological grading and cell viability results revealed that the porcine SIS mesh exhibited slight cytotoxicity. For RTCA method, as shown in Fig. 4F, after cells were seeded into 8 wells E-plate, the CI curve within 24 h represents the process of cell adherence and proliferation and the fluctuation of the CI values was observed in L929 cell lines cultured with extracts of SIS and composite mesh. According to the CI value, SIS extracts show slow downtrend to the tested cell lines. In contrast, the composite mesh extracts drop significantly at 58 h. The results of RTCA produced similar results to MTT.

### Results of chemotaxis assays

**Figure 5:**
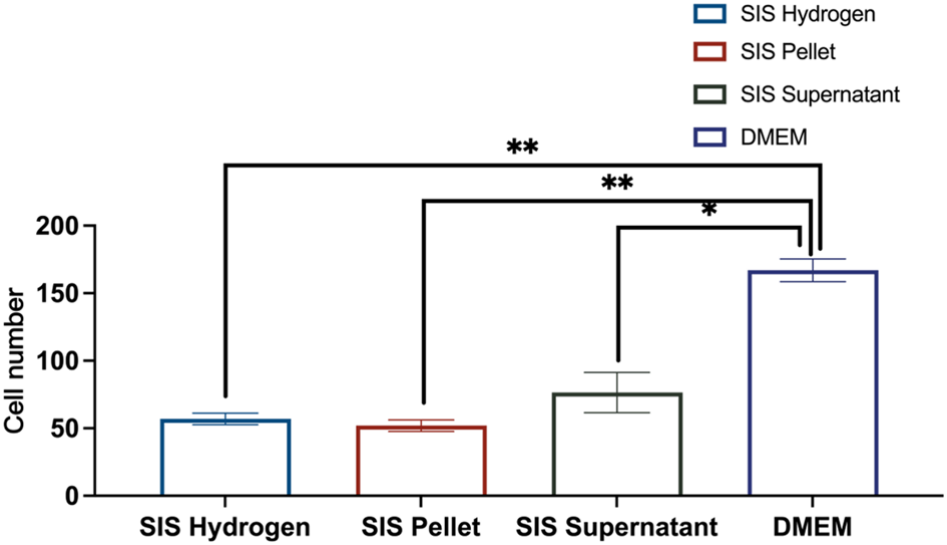
Boyden Chamber chemotaxis assays were performed in vitro using SIS Hydrogen, SIS Pellet and SIS Supernatant. Bal/b/c 3T3 cells were set up to migrate through the filter towards three different SIS products and imaging. All the three SIS products could significantly inhibit the chemotactic activity of Bal/b/c 3T3 cells. The decrease in cellular chemotaxis ranged from a 2–3 fold compared with DMEM control (p < 0.05). No significant differences were found between different SIS products (p > 0.05).

As shown in Fig. 5, the average cell numbers that migrated through the filter of all three different SIS products was significantly decreased over DMEM control. The decrease in cellular chemotaxis ranged from a 2–3 fold compared with DMEM control (p < 0.05). Meanwhile, there is no significant differences were found between different SIS products (p > 0.05).

### Results of implantation and degradation in vivo

**Figure 6:**
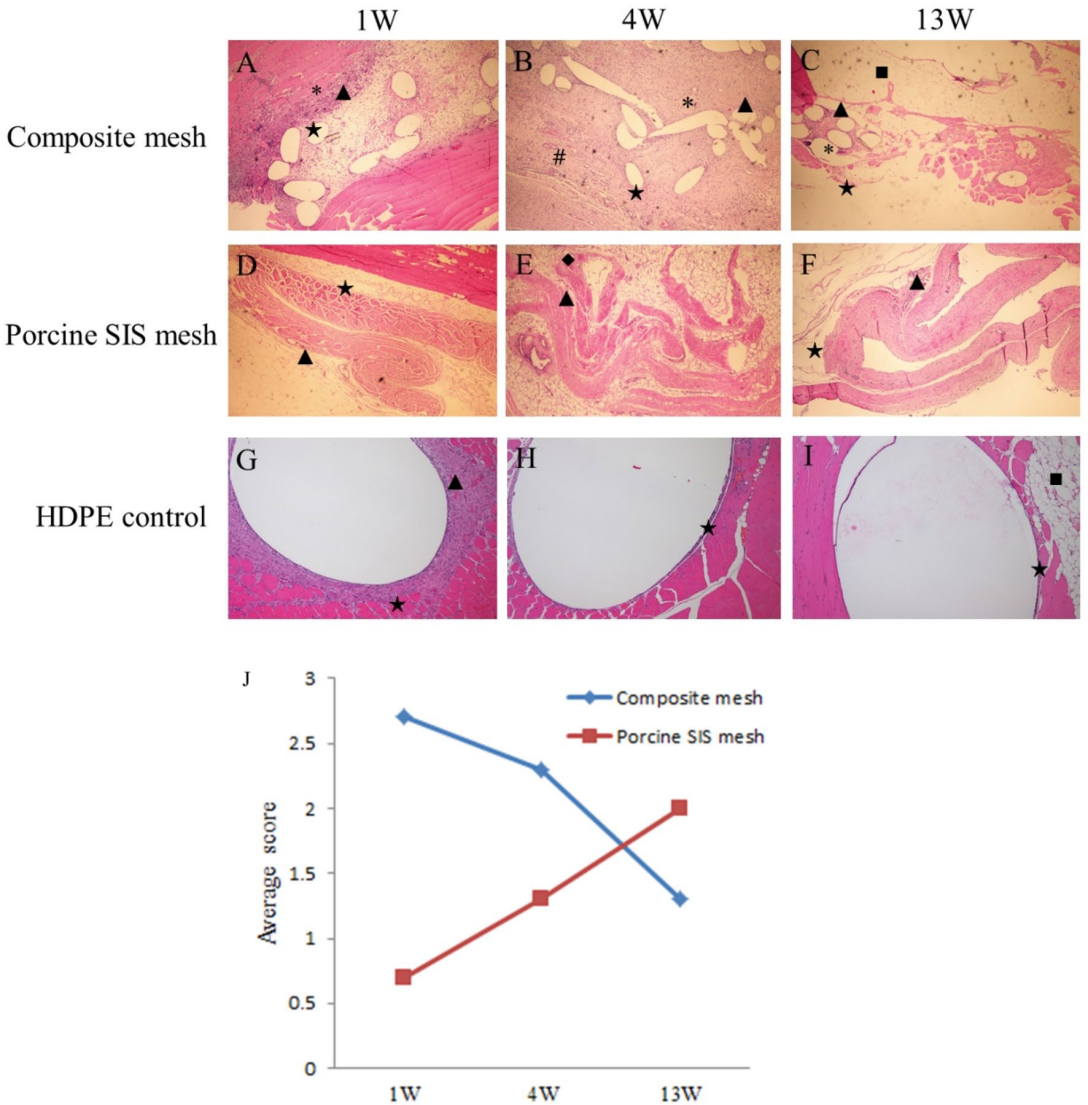
Results of local reaction and degradation of the composite mesh and porcine SIS mesh (40 × magnification). (**A**–**C**) 1 w, 4 w and 13 w after the composite mesh implantation, (**D**–**F**) 1 w, 4 w and 13 w after the SIS mesh implantation, (**G**–**I**) 1 w, 4 w and 13 w after the HDPE implantation, (**J**) the average score for the composite mesh and the SIS mesh based on the semi-quantitative scoring scheme. Macrophage (▲), Polymorphonuclear cells (*), Fibroblast (★), Lymphocytes (◆), Fatty infiltration (■), Neovascularisation (#).

As shown in Fig. 6, the macroscopic reaction in different groups was normal for 1 week, 4 weeks and 13 weeks after rabbit subcutaneous implantation. One week after implantation, the average score between the composite mesh and the negative control was 2.7, while the Porcine SIS mesh was 0.7 using the H&E stain and microscopic semi-quantitative scoring scheme. 4 weeks after implantation, the average score between the composite mesh and the negative control was 2.3. However, the Porcine SIS mesh was 1.3. 13 weeks after implantation, the average score difference between the composite mesh and the negative control was 1.3, while the Porcine SIS mesh was 2.0. Semi-quantitative scoring results indicated that the porcine SIS mesh displayed accumulated characteristics of minimal inflammatory reaction, while there was a descending inflammatory reaction in the composite mesh group. Compared to the composite mesh (Fig. 6A–C), porcine SIS mesh implants displayed inflammatory cell infiltration as well as collagen distribution alteration and mild degradation over time (Fig. 6D–F).

### Establishment of experimental porcine hernia model

After 90 d of animal model preparation, obvious hernia content palpation occurred in all animal models. As shown in Table 3, the hernia ring formation rate of different groups indicating the experimental porcine model of ventral hernia repair has been successfully established.

**Table 3:** Hernia ring formation rate of different groups (mean ± SD).

Groups (n = 6)	Hernia ring diameter (mm)		Formation rate (%)
	Length	Width	
Composite mesh	104 ± 8	62 ± 18	100
Porcine SIS mesh	107 ± 18	59 ± 16	100

### Adhesion evaluation

**Figure 7:**
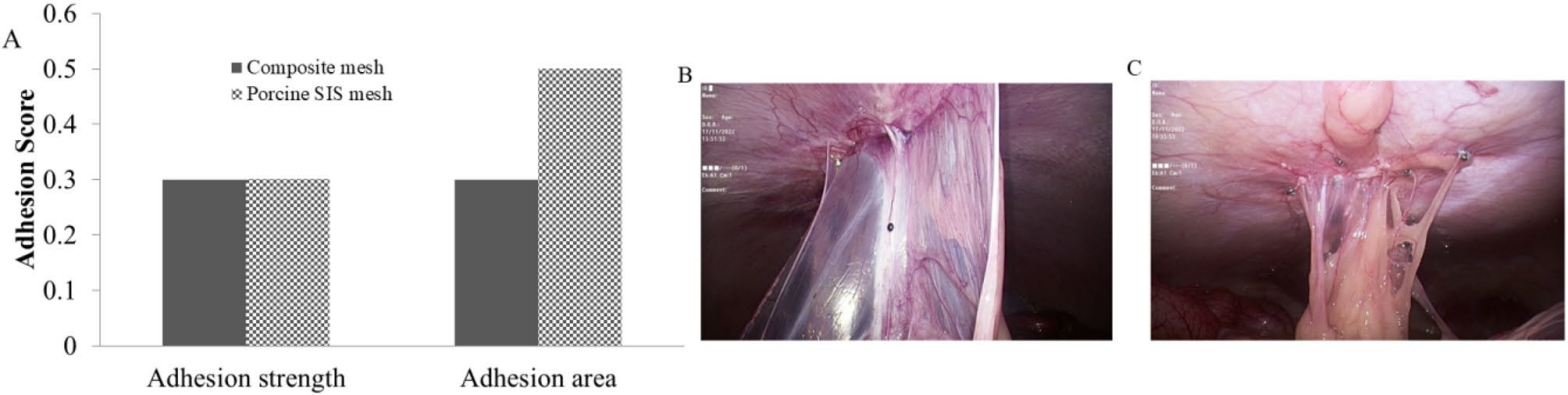
Results of adhesion formation on the composite mesh and porcine SIS mesh. (**A**) Average adhesion score based on strength and area, macroscopic appearance of adhesion formation with (**B**) porcine SIS mesh, and (**C**) composite mesh (n = 6 per group).

At 1 month, porcine intra-peritoneal adhesions of the composite mesh and porcine SIS mesh were recorded and scored according to the reported adhesion scoring criteria. As shown in Fig. 7, infrequent adhesion development occurred in the nailed part both in the composite and porcine SIS mesh under laparoscopic observation, which showed good anti-adhesion effect and excellent tissue compatibility of the meshes.

### Mechanical testing

**Figure 8:**
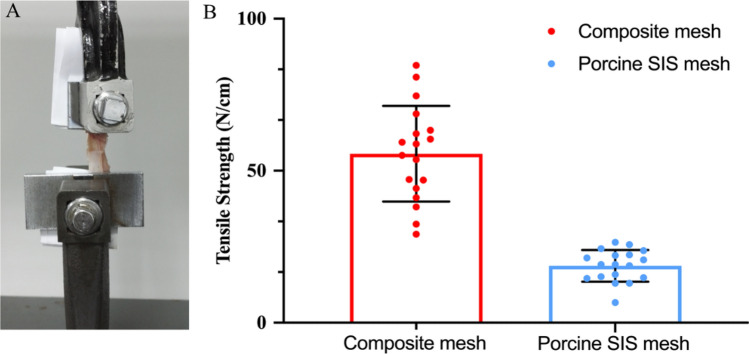
Mechanical properties of the composite mesh compared to the porcine SIS mesh, (**A**) measurement of tensile strength in the universal testing machine and (**B**) relative stiffness (N/cm). *p < 0.05. The data presented here represent a total of three independent replicates.

The results for the tensile strength of the composite mesh and the porcine SIS mesh explant were given in Fig. 8A,B, that is, 55.6 N ± 18.7 N for the composite mesh and 15.7 N ± 5.2 N for the porcine SIS mesh. There was a significant difference between the two meshes (p < 0.05).

### Histologic analysis

**Figure 9:**
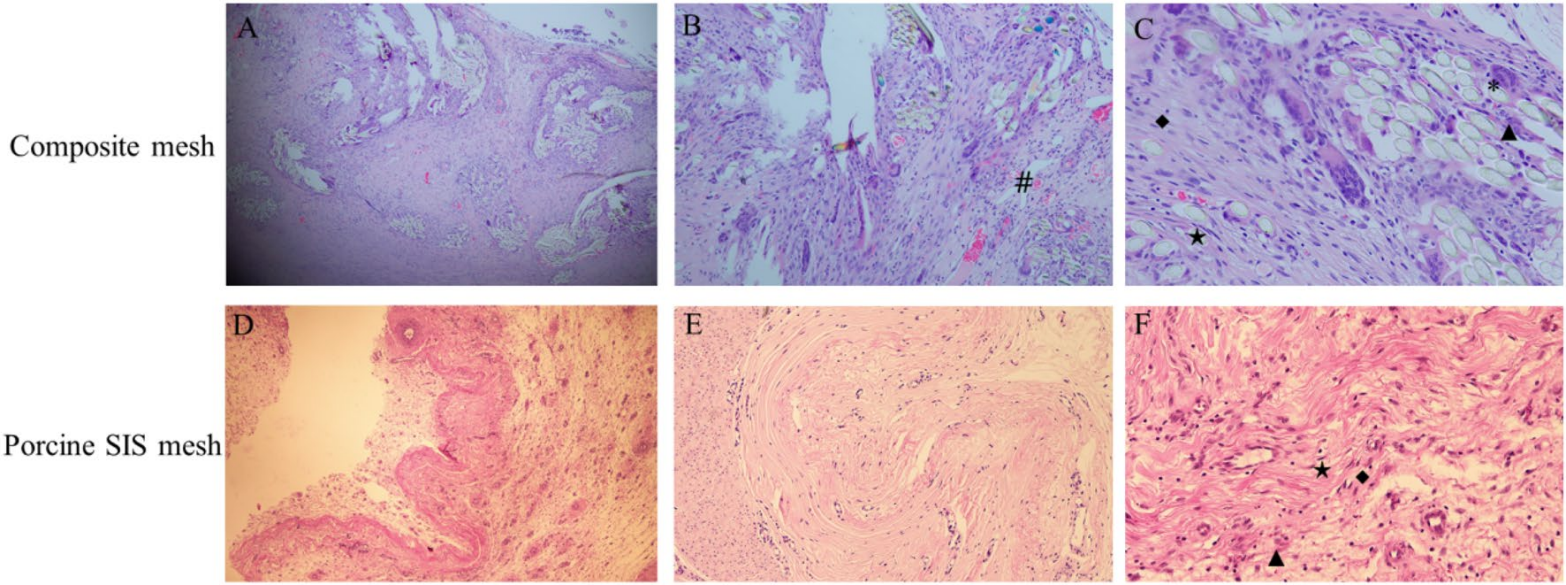
Histologic appearance of the host tissue response to meshes with H&E photomicrographs at 30 d after implantation. Composite mesh: (**A**) 40 × magnification, (**B**) 100 × magnification, and (**C**) 200 × magnification; porcine SIS mesh: (**D**) 40 × magnification, (**E**) 100 × magnification, and (**F**) 200 × magnification. Polymorphonuclear cells (*), Macrophages (▲), Lymphocytes (◆), Fibroblast (★), Neovascularisation (#).

From Fig. 9A–F, the histologic analysis showed that the porcine SIS mesh displayed prominent inflammatory cellular infiltration compared to the composite mesh, including macrophages, multinucleated giant cells, lymphocytes and neovascularization reactions after implantation of 30 days according to the established semi-quantitative scoring system. In addition, both meshes displayed an excellent tissue ingrowth capacity. Also, the two groups have no significant difference in fatty infiltration and fibrosis. No significant scaffold degradation was identified in the two groups, as well as sparse host ECM deposits on the mesh surface. According to the scoring system we have established for evaluating tissue remodeling and regeneration, the average score of the composite mesh is 10, while 9.9 is for the porcine SIS mesh, as seen in Table 4.

**Table 4:** Semi-quantitative scoring system for evaluating tissue remodeling and regeneration.

Response	Composite mesh	Porcine SIS mesh
Integration	40	40
Fatty infiltration	0	0
Neovascularisation	4	8
Fibrosis	7	6
Cellular infiltration	10	12
Tissue ingrowth	26	22
ECM deposition	13	11
Scaffold degradation	0	0
Total	100	99
Average	10	9.9

## Discussion

A variety of composite meshes and porcine ECM meshes have been available for clinical applications^27–30^. Porcine SIS meshes were prepared from the porcine small intestine submucosa tissue by decellularization process. Thus, the recognized components that remained are collagen, growth factors, glycoproteins and some cellular material^31^. Though these newly developed meshes, in combination with a laparoscopic method, have reduced recurrence rates and other complications, different researchers have argued how to fully characterize the remained protein as well as immunogenic residual and how to balance the active component mediated inflammation reaction and tissue remodeling effect, especially for some commercial porcine SIS meshes over the past decades^32,33^.

It should be noted that biomechanical and histologic analyses have been utilized in different animal models to evaluate the properties of meshes over the past few years^34,35^. However, there are still obscure to develop an ideal animal model to evaluate the remodeling performance of biological meshes, which would possess several characteristics, including reproducible, measurable, quantitative and applicable to clinical scenarios. Therefore, establishing an ideal animal repair model is crucial in evaluating meshes' effectiveness and safety.

In this study, we successfully prepared a kind of novel SIS mesh derived from submucosal tissue of the porcine small intestine and determined the microstructure using SEM, since it is essential for biocompatibility and tissue repair efficacy. As shown in Fig. 1, our results demonstrated that the surface of the SIS mesh exhibits an obvious irregular microstructure with overlapping collagen. This microscopic result would improve organic tissue cell ingrowth and differentiation after mesh implantation and promote wound repair and tissue remodeling.

Regarding the residual DNA of the SIS mesh and α-Gal antigen clearance study, our results indicated that the amount of residual DNA in the SIS mesh was 344.24 ± 6.06 ng/mg, while the clearance of α-gal antigen was 54.01% ± 0.02%. Both of these findings demonstrate that the process we used to prepare SIS meshes cannot wholly remove the cellular components and active components of the ECM that might be responsible for the minor local inflammatory response after implantation. However, at the same time, these were also essential factors for SIS meshes to perform some function in promoting tissue remodeling while achieving the optimal balance state between the two requires further in-depth studies. Subsequently, we employed in vitro cell tests, Bal/b/c 3T3 cell chemotaxis assay and in vivo implantation experiments in rabbits to investigate the biocompatibility of SIS meshes. As shown in Figs. 4 and 5, the cytotoxicity assay results showed that SIS meshes had slight cytotoxic effects and the decrease in cellular chemotaxis ranged from a 2–3 fold compared with DMEM control, indicating the components in SIS meshes could inhibit cell chemotaxis to play a key role in the development of tissue remodeling. In contrast, the results of subcutaneous implantation in rabbits showed that the SIS mesh presented a local minor inflammatory response dominated by macrophage and lymphocyte infiltration, which was considered to be related to the residual cellular components and active components in the meshes (Fig. 6).

We have constructed a porcine ventral hernia surgical model using the laparoscopic with open hybridization method in the present study. As shown in Table 2, the rate of hernia ring formation was 100% in animals of different groups. We then used this model to evaluate the efficacy, safety and tissue remodeling capability of the prepared acellular SIS meshes. As shown in Fig. 7, one month after hernia repair surgery, the formation of few tissue adhesions was found only at the mesh fixation site under laparoscopic observation for the composite mesh and SIS meshes, indicating that both had better anti-adhesion effect and histocompatibility. The results of the mechanical test show that, as shown in Fig. 8, the mechanical effect of the composite meshes containing polyester materials is better than that of the SIS meshes containing only extracellular matrix materials, consistent with most of the reported results^36,37^.

In the tissue remodeling and scoring system study, as shown in Table 4, the neovascularization and cellular infiltration were significantly increased in the tissue reaction of SIS meshes compared with the composite meshes, which was related to the cellular component residues and active components contained in SIS meshes and also predicted its pronounced effect of promoting tissue remodeling. At the same time, organismal integration, tissue ingrowth and ECM deposition occurred in both meshes, indicating better histocompatibility, supporting tissue remodeling at later stages. In addition, the degradation of SIS meshes was not observed in this study due to the short mesh implantation time, and we will focus our subsequent studies on the degradation process of SIS meshes.

## Conclusion

In summary, we developed a useful semi-quantitative scoring system in a porcine hernia repair model that can be used for the preclinical safety and efficacy evaluation of meshes. In addition, our study has demonstrated that the component of SIS mesh could regulate cell behavior to exhibit the function during tissue remodeling in the experimental animal model. The following investigation will focus on more detailed cellular responses with material components including pro-inflammatory factors and signaling pathway during ECM mesh application.

## Data Availability

The datasets used and/or analysed during the current study available from the corresponding author on reasonable request.

## References

[1] Sawyer M, Ferzoco S, DeNoto G (2020). Hernia mesh and hernia repair: A review. Eng. Regen..

[2] Kalaba S, Gerhard E, Winder JS, Pauli EM, Haluck RS, Yang J (2016). Design strategies and applications of biomaterials and devices for Hernia repair. Bioact. Mater..

[3] Breuing KH, Colwell AS (2007). Inferolateral AlloDerm hammock for implant coverage in breast reconstruction. Ann. Plast. Surg..

[4] Cheng CW, Solorio LD, Alsberg E (2014). Decellularized tissue and cell-derived extracellular matrices as scaffolds for orthopaedic tissue engineering. Biotechnol. Adv..

[5] Elango S, Perumalsamy S, Ramachandran K, Vadodaria K (2017). Mesh materials and hernia repair. Biomedicine (Taipei).

[6] Kingsnorth A (2004). Treating inguinal hernias: Open mesh Lichtenstein operation is preferred over laparoscopy. Br. Med. J..

[7] Whitehead-Clarke T, Karanjia R, Banks J (2022). The experimental methodology and comparators used for in vivo hernia mesh testing: A 10-year scoping review. Hernia.

[8] Collins MM, Race B, Messer RJ (2023). Practical mouse model to investigate therapeutics for *Staphylococcus*
*aureus* contaminated surgical mesh implants. J. Surg. Res..

[9] Crapo PM, Gilbert TW, Badylak SF (2011). An overview of tissue and whole organ decellularization processes. Biomaterials.

[10] Hussey GS, Dziki JL, Badylak SF (2018). Extracellular matrix-based materials for regenerative medicine. Nat. Rev. Mater..

[11] Heath DE (2019). A review of decellularized extracellular matrix biomaterials for regenerative engineering applications. Regen. Eng. Transl. Med..

[12] Mao Y, Meng Y, Li S, Li Y, Guidoin R, Qiao Y (2021). Comparative study on nanofiber containing polypropylene-based composite mesh for abdominal wall hernia repair. Mater. Des..

[13] Gaertner WB, Bonsack ME, Delaney JP (2010). Visceral adhesions to hernia prostheses. Hernia.

[14] Chatzimavroudis G, Kalaitzis S, Voloudakis N, Atmatzidis S, Kapoulas S, Koutelidakis I, Papaziogas B, Christoforidis EC (2017). Evaluation of four mesh fixation methods in an experimental model of ventral hernia repair. J. Surg. Res..

[15] Wiegering A, Schlegel N, Isbert C, Jurowich C, Doht S, Germer CT, Dietz UA (2013). Lessons and challenges during a 5-year follow-up of 21 Composix Kugel implantations. Hernia.

[16] Patiniott P, Stagg B, Karatassas A, Maddern G (2020). Developing a hernia mesh tissue integration index using a porcine model—A pilot study. Front. Surg..

[17] Zheng MH, Chen J, Kirilak Y, Willers C, Xu J, Wood D (2005). Porcine small intestine submucosa (SIS) is not an acellular collagenous matrix and contains porcine DNA: Possible implications in human implantation. J. Biomed. Mater. Res. B Appl. Biomater..

[18] Casarin M, Fortunato TM, Imran S, Todesco M, Sandrin D, Borile G, Toniolo I, Marchesan M, Gerosa G, Bagno A, Romanato F, Carniel EL, Morlacco A, Dal Moro F (2022). Porcine small intestinal submucosa (SIS) as a suitable scaffold for the creation of a tissue-engineered urinary conduit: Decellularization, biomechanical and biocompatibility characterization using new approaches. Int. J. Mol. Sci..

[19] Qiu S, Liang L, Zou P, Chen Q (2021). Decellularized small intestine submucosa/polylactic-co-glycolic acid composite scaffold for potential application in hypopharyngeal and cervical esophageal tissue repair. Regen. Biomater..

[20] Raeder RH, Badylak SF, Sheehan C, Kallakury B, Metzger DW (2002). Natural anti-galactose alpha1,3 galactose antibodies delay, but do not prevent the acceptance of extracellular matrix xenografts. Transpl. Immunol..

[21] Vogels RRM, Kaufmann R, van den Hil LCL (2017). Critical overview of all available animal models for abdominal wall hernia research. Hernia.

[22] Gai X, Liu C, Wang G, Qin Y, Fan C, Liu J, Shi Y (2020). A novel method for evaluating the dynamic biocompatibility of degradable biomaterials based on real-time cell analysis. Regen. Biomater..

[23] Vlahos A, Yu P, Lucas CE, Ledgerwood AM (2001). Effect of a composite membrane of chitosan and poloxamer gel on postoperative adhesive interactions. Am. Surg..

[24] Novotný T, Jeřábek J, Veselý K, Staffa R, Dvořák M, Cagaš J (2012). Evaluation of a knitted polytetrafluoroethylene mesh placed intraperitoneally in a New Zealand white rabbit model. Surg. Endosc..

[25] Diamond MP, Linsky CB, Cunningham T, Constantine B, diZerega GS, DeCherney AH (1987). A model for sidewall adhesions in the rabbit: Reduction by an absorbable barrier. Microsurgery.

[26] Hollinsky C, Kolbe T, Walter I, Joachim A, Sandberg S, Koch T, Rülicke T, Tuchmann A (2010). Tensile strength and adhesion formation of mesh fixation systems used in laparoscopic incisional hernia repair. Surg. Endosc..

[27] Hu X, Cebe P, Weiss AS, Omenetto F, Kaplan DL (2012). Protein-based composite materials. Mater. Today.

[28] Poussier M, Denève E, Blanc P (2013). A review of available prosthetic material for abdominal wall repair. J. Visc. Surg..

[29] Alexandridis V, Teleman P, Rudnicki M (2021). Efficacy and safety of pelvic organ prolapse surgery with porcine small intestinal submucosa graft implantation. Eur. J. Obstet. Gynecol. Reprod. Biol..

[30] Basonbul RA, Cohen MS (2017). Use of porcine small intestinal submucosa for pediatric endoscopic tympanic membrane repair. World J. Otorhinolaryngol. Head Neck Surg..

[31] Luo JC, Chen W, Chen XH, Qin TW, Huang YC, Xie HQ, Li XQ, Qian ZY, Yang ZM (2011). A multi-step method for preparation of porcine small intestinal submucosa (SIS). Biomaterials.

[32] Moroni F, Mirabella T (2014). Decellularized matrices for cardiovascular tissue engineering. Am. J. Stem Cells.

[33] Knoll LD (2007). Use of small intestinal submucosa graft for the surgical management of Peyronie’s disease. J. Urol..

[34] Liu L, Li D, Wang Y, Xu H, Ge L, Liang Z (2011). Evaluation of the biocompatibility and mechanical properties of xenogeneic (porcine) extracellular matrix (ECM) scaffold for pelvic reconstruction. Int. Urogynecol. J..

[35] Stoikes NFN, Scott JR, Badhwar A, Deeken CR, Voeller GR (2017). Characterization of host response, resorption, and strength properties, and performance in the presence of bacteria for fully absorbable biomaterials for soft tissue repair. Hernia.

[36] Jenkins ED, Melman L, Deeken CR, Greco SC, Frisella MM, Matthews BD (2011). Biomechanical and histologic evaluation of fenestrated and nonfenestrated biologic mesh in a porcine model of ventral hernia repair. J. Am. Coll. Surg..

[37] Jenkins ED, Melman L, Deeken CR (2010). Evaluation of fenestrated and non-fenestrated biologic grafts in a porcine model of mature ventral incisional hernia repair. Hernia.

